# Effects of a novel, non-invasive pre-hatch application of probiotic for broilers on development of cecum microbiota and production performance

**DOI:** 10.1186/s42523-023-00263-7

**Published:** 2023-09-05

**Authors:** Kasper Rømer Villumsen, Dorthe Sandvang, Gisle Vestergård, Mia Son Räfle Olsen, Johanne Juul, Morten Dencker, Johannes Kudsk, Louise Ladefoged Poulsen

**Affiliations:** 1 Department of Veterinary and Animal Sciences, Faculty of Health and Medical Sciences, University of Copenhagen, Stigboejlen 4, 1870 Frederiksberg, Denmark; 2 Chr. Hansen A/S, Animal Health Innovation, Boege Allé 10, Hoersholm, Denmark; 3 Ceva Animal Health, Ladegårdsvej 2, 7100 Vejle, Denmark; 4 DanHatch A/S, Hoertoftvej 14, 6400 Soenderborg, Denmark

**Keywords:** Broiler production, Broiler performance, Field trial, Cecal microbiota, Early probiotic application

## Abstract

**Background:**

Probiotics are used in the broiler industry to increase production performance. Most often a probiotic is applied by mixing it in the feed, but studies have shown that earlier application may be advantageous. Therefore, *in ovo* application where the probiotic is administrated into the egg before hatch has been investigated as an alternative application method. However, *in ovo* application may impact hatchability negatively and may not be feasible at all hatcheries. The purpose of this study was to investigate the effect of a novel non-invasive method for mass application before hatch. The probiotic (*E. faecium* 669*)* was applied as a single dose by spray on the unhatched eggs and production performance and development of the cecal microbiota until slaughter was compared with a control flock. Through 16S rRNA sequencing of cecal samples from 25 broilers at day 7, 21 and 37 we compared the microbiota composition and richness for each group. The study was repeated for additional recording of production performance and re-isolation of the probiotic *E. faecium* from the intestine.

**Results:**

In both trials the probiotic *E. faecium* could be re-isolated from the yolk sac and intestine at hatch and at day 7. Broilers in the probiotic treated groups had a higher performance in terms of bodyweight at day 34 and European production efficiency factor. Finally, a significant reduction of first-week and overall mortality was observed in the probiotic group in the first trial. Based on 16S rRNA profiling, significant differences in alpha diversity were found exclusively at day 37. Estimation of beta diversities, however, identified significant differences in microbiota composition between the control and probiotic group at day 7, 21 and 37.

**Conclusion:**

The probiotic *E. faecium* strain successfully colonized broilers before/during hatch after a single spray application at day 18 of incubation. Positive effects of the probiotic were observed in multiple production parameters, including reduced mortality in trial 1, and microbiota analyses indicate significantly different microbiota compositions throughout the experimental phase. Taken together, the novel low-tech mass administration of *E. faecium* (669) may be considered a feasible strategy for improvements of production parameters in broiler production.

**Supplementary Information:**

The online version contains supplementary material available at 10.1186/s42523-023-00263-7.

## Background

The poultry industry is important for global food security and optimizing the production in regard of efficient production and better welfare is of high priority. The first week of life is a transition period where the newly hatched chickens need to adapt to changes. They change from utilizing the yolk sac as nutrition to digestion of feed and water, and the immune system starts to mature. First week mortality (FWM) is a key parameter showing how well the broilers adapt to the new environment and is affected by both internal and external factors [1, 2]. Internal factors as breeder age, chick gender and breed affect FWM [3]. External factors as the type of broiler house, presence or absence of drip cups, egg storage and season have also been identified as factors which impacts FWM [3]. The first week accounts for approximately 20% of the total lifetime and in a Norwegian study it was found that in this period the average cumulative mortality was 1.54% while the remaining period only accounts for only 0.48% of the cumulative mortality [4]. Yassin and colleagues (2009) found that FWM ranged from 0 to 3.3% but again with an average of 1.5%. Such data indicates that early intervention may be beneficial in terms of decreasing mortality. A previous study has shown that *in ovo* application of the specific probiotic strain was advantageous in terms of improving production performance and intestinal morphology in broilers [5]. However, *in ovo* injection may impact hatchability negatively and may not be applicable at all hatcheries. To address the issue of pre-hatch delivery of probiotics, we investigated a new non-invasive application method for broilers where the probiotic *E. faecium* 669 was sprayed on the eggs at day 18 of incubation. The eggs were sprayed during transition from the setters to the hatchers when eggs were handled for other reasons. Previous studies have found that probiotic has the potential to stimulate a beneficial microbiota and hereby contribute to increased health of the host [6]. In support of this aim, an association between body weight and intestinal microbiota composition has been described by Lundberg et al. [7]. It was demonstrated that high body weight at time of slaughter was correlated with high alpha-diversity, high levels of short-chain-fatty-acids-producing and health associated bacterial taxa in ceca. Another study demonstrated that application of host-tailored probiotics can alter the developing microbiota in turkeys [8]. These studies indicate that probiotics may be a tool to alter the microbiota to a composition that favours increased productivity. Often probiotics are continuously applied through the feed but in this study, we investigate if a single dose of probiotic *E. faecium* (669) sprayed on the unhatched eggs at day 18 of incubation impacts production parameters and the development of the cecal microbiota composition of broilers. Like a butterfly effect with one small event affecting a large and complex system.

## Results

### Probiotic strain

The actual concentration of the sprayed solution was 5.8 × 10^8^ and 3.3 × 10^8^ colony forming units (cfu) per egg in trial 1 and 2, respectively.

### Hatchability

Hatchability in trial 1 were 84,4% in both the probiotic and the mock-sprayed eggs and in trial 2, hatchability was 77,6% in both groups.

### Recovery of Probiotic bacteria at day 1, 7 and 21

In trial 1 the recovery of the probiotic *E. faecium* from yolk-sac and intestine at day 1 and cecum samples at day 7 and 21 showed that the probiotic was present in the cecum at hatch in 96% of the chickens and declined to 48% at day 7. At day 21 the probiotic *E. faecium* could not be detected by the method used. In the second trial the probiotic strain could only be recovered from 64% of the newly hatched chickens and 40% were positive at day 7. Samples from day 21 were not analyzed in trial 2 (Table 1).

**Table 1:** Recovery of *E. faecium* from broilers in trial 1 and 2

Day	Trial 1			Trial 2		
	1	7	21	1	7	21
% of treated birds	96	48	0	64	40	ND*

The control flocks in trial 1 and 2 were sampled at the same time as the probiotic treated flocks. At no time point the *E. faecium* were recovered from the control flocks (data not shown in the table).

*Not determined.

**Fig. 1:**
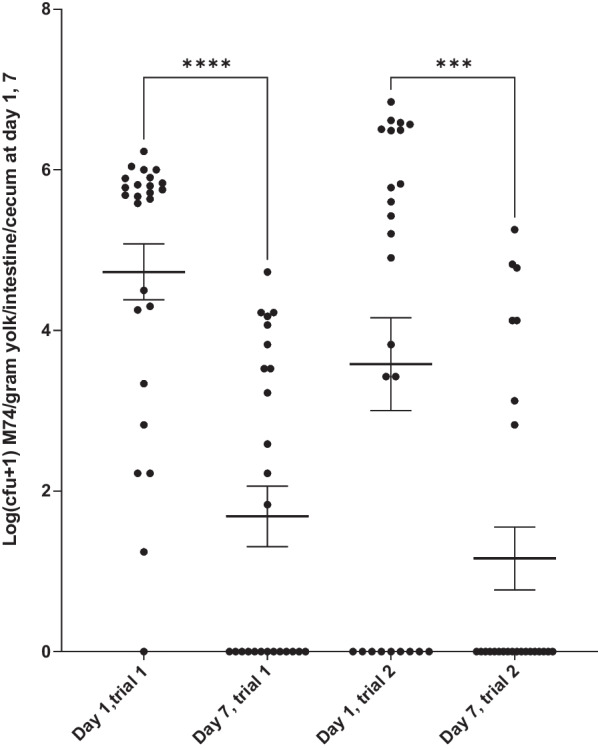
The first two scatter graphs show data from trial 1 and the last two scatter graphs show data from trial 2. Mean log(cfu + 1) *E. faecium* per gram of yolk-sac and intestine (day 1) and cecum (day 7). Ordinary one-way ANOVA, Mean with Standard error of mean is shown. Significant differences are indicated by asterisks as follows: *** = *P* < 0.001 and **** = *P* < 0.0001. On the graph only differences within each trial are shown

The cfu of *E. faecium* probiotic strain per gram intestine/yolk-sac (ceca at day 7) in chickens at day 1 and 7 is shown in Fig. 1. The probiotic bacteria were not found in the control group at any time points. The concentration of the probiotic declined significantly from day 1 to day 7 in both trials (*P* < 0.0001, trial 1 and *P* = 0.0009, trial 2). The cfu values were higher in the first trial compared to the second trial, however, statistically no difference could be detected. At day 1 the mean log cfu of the probiotic strain was not significantly different in trial 1 compared to trial 2 (*P* = 0.2463) and no significant difference could be detected at day 7 either (*P* = 0.8283) (ordinary one-way ANOVA). The cfu varies considerably between individuals and at day seven, 13 and 18 samples were negative for the probiotic resulting in the low mean cfu values at day 7.

### Production performance

**Fig. 2:**
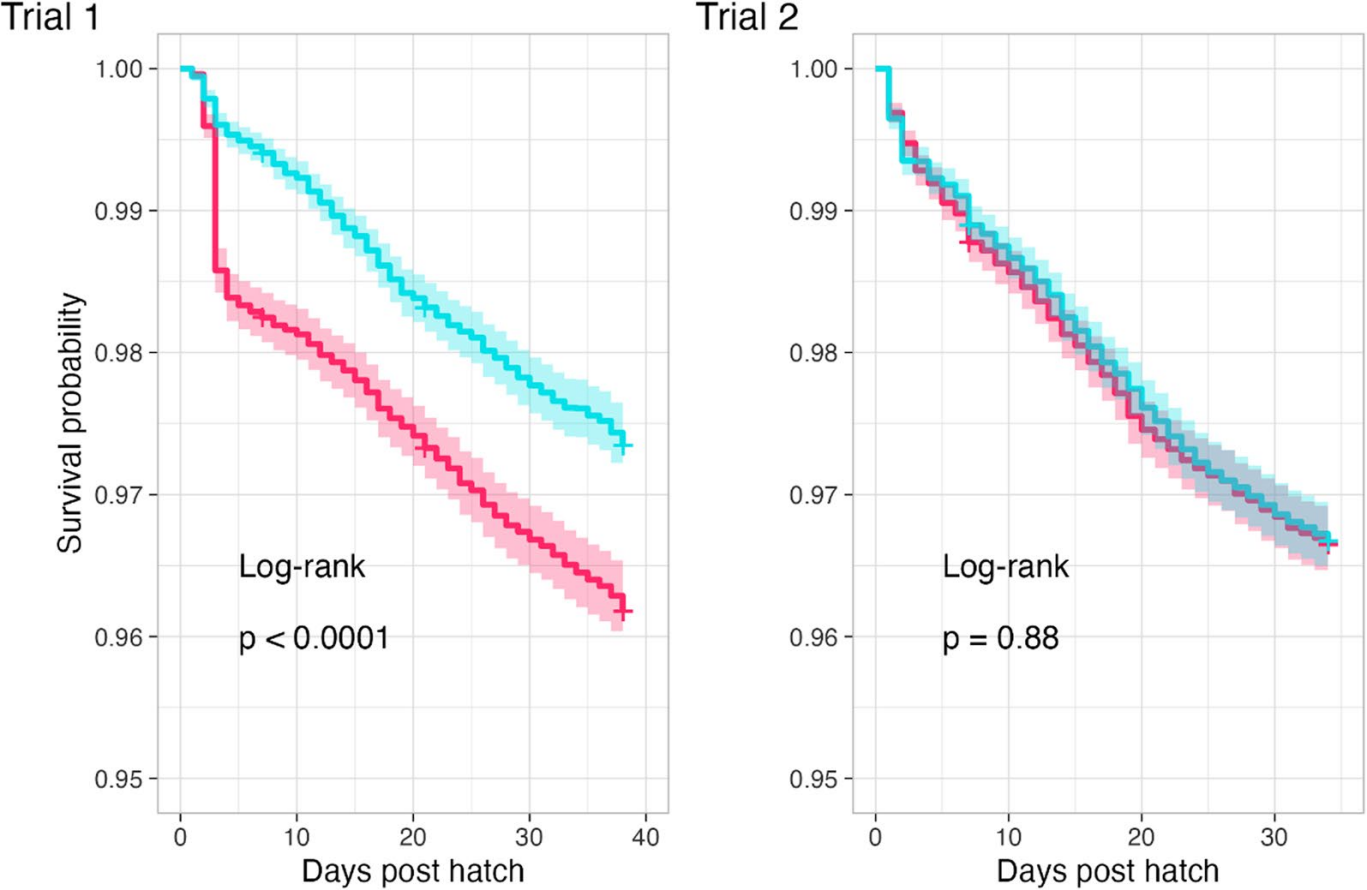
Kaplan Meier curves illustrating the mortality in trials 1 and 2. Time in days at the x-axis and survival probability at the y-axis. Each curve is shown with its 95% confidence interval

**Fig. 3:**
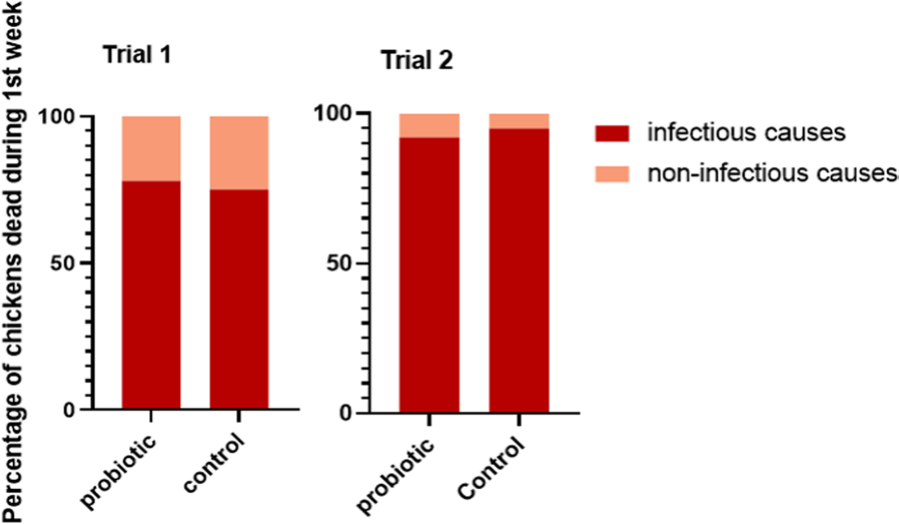
The first bar graph shows data from trial 1 and the second bar graph shows data from trial 2. Percentage of chickens which died during first week of life due to infectious or non-infectious causes, based on *post mortem* examination and bacteriological culturing in trial 1 and trial 2

Table 2 summarizes production data. It is found that in trial 1, first week mortality (FWM) and the total mortality was significant lower in the probiotic treated group compared to the control group. However, in the second trial no difference in either FWM or total mortality were found. From the Kaplan Meier curve (Fig. 2, trial 1) it is illustrated that the difference in mortality in trial 1 is caused by a high mortality at day three where 224 chickens die. No known incidences at the farm could explain the high mortality at day three. *Postmortem* investigation was performed for all collected chickens that died during first week of life (208 and 406 chickens were collected in trial 1 and 2, respectively). In trial 1, it was found that 78% and 75% of the dead chickens in the probiotic and control flock, respectively, died due to infectious causes (*P* = 0.5929) (Fig. 3A). *E. coli* was isolated from most chickens, which were positive for culturing; 94% and 81% from the control and probiotic group, respectively. However, from 60% of the chickens in the control group and 38% in the probiotic group which showed lesions in compliance with bacterial infection bacteria could not be cultured. In the second trial the first week mortality was 1.4% and 1.2% in the probiotic and control flock, respectively. In this trial 92% and 95% of the dead chickens in the probiotic and control flock, respectively, died due to infectious causes (*P* = 0.6169) (Fig. 3B), again most died due to *E. coli* infections (*E. coli* was isolated from 88 and 66% of all samples positive for culturing in the probiotic and control group, respectively).

**Table 2:** Production data from trial 1 and 2 with comparison to Ross performance objectives as hatch

	Trial 1			Trial 2			Ross 308 Performance objectives (as hatch) [9]
	Control	Probiotic	*p*-value	Control	Probiotic	*p*-value	
Flock size	22,000	21,700		24,100	24,200		NA
Breeder age (weeks)	28	28		60	60		NA
First week mortality %	1.9^a^	0.5^b^	< 0.0001	1.2	1.4	0.1009	NA
Total mortality %	3.8^a^	2.4^b^	< 0.0001	3.3	3.6	0.0656	NA
FCR	1.42	1.39		1.39	1.38		1.38
EPEF	411	448		436	446		NA
Bodyweight day 7 (grams)	185	187		205	205		213
Body weight day 34 (grams)	2061	2173		2140	2178		2196

*NA* Not available

^a, b^significantly different from each other

FCR differs slightly between the two groups in both trial 1 and 2 (0.03 and 0.01 lower in the probiotic treated group in trial 1 and 2, respectively). At day 34 the average bodyweight was 112 g (5.4%) higher in the probiotic treated group compared to broilers in the control group in trial 1. Because of the lower mortality and higher bodyweight, the European Production Efficiency Factor (EPEF) ends up being 9% higher in the probiotic treated group in the first experiment. In trial 2 the body weight and EPEF was 1.8% and 2.3% higher in the probiotic treated group compared to the control group. However, the FWM was slightly higher in the probiotic treated broilers with a mortality of 1.4% compared to 1.2% in the control group.

### Microbial community analysis of ceca

**Fig. 4:**
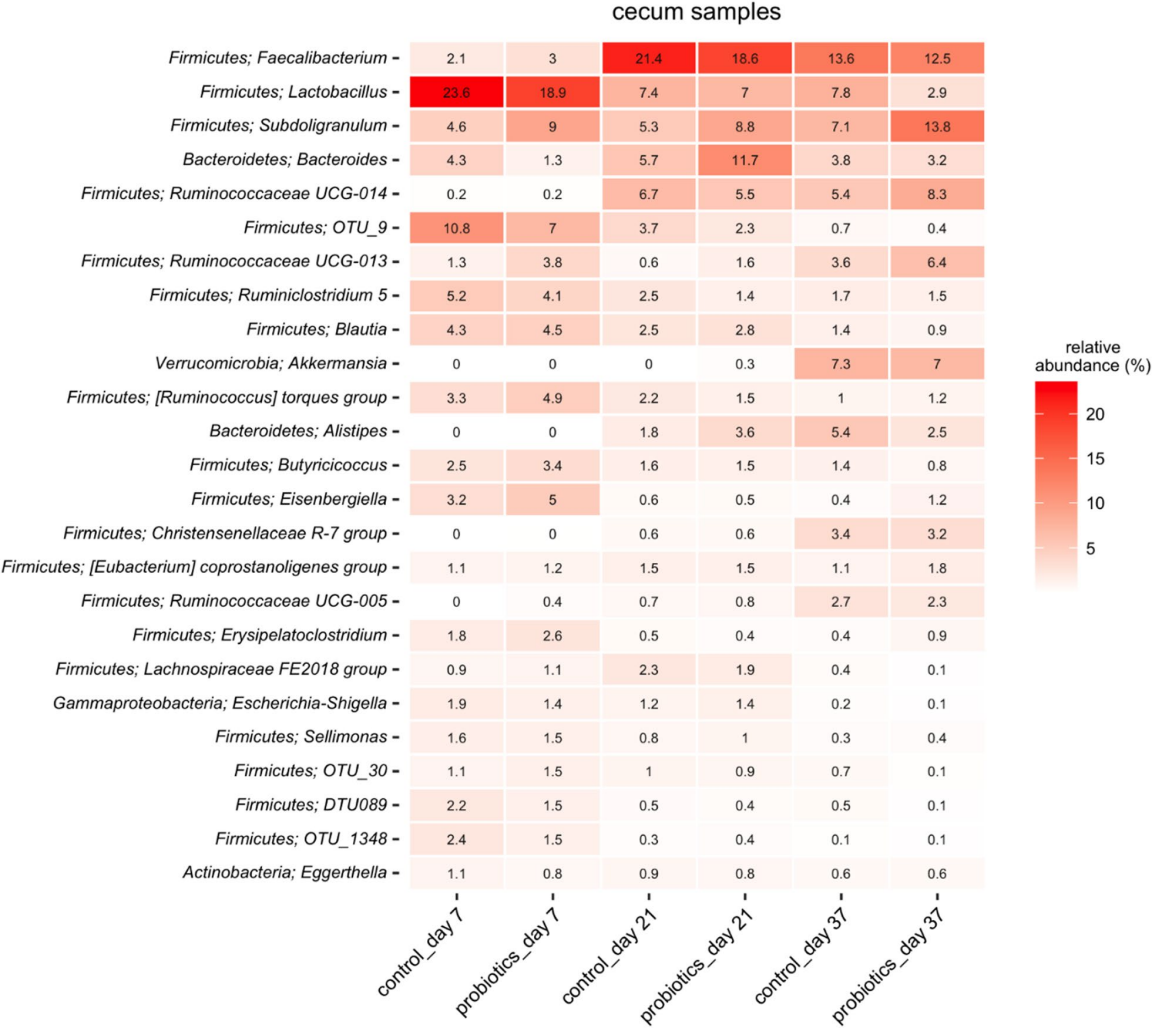
Heatmap showing relative OTU abundances for control- and probiotic group in trial 1, day 7, 21 and 37

Potential effects of the probiotic treatment on cecal microbiota structure during trial 1 were addressed through 16S rRNA sequencing. Figure 4 shows the 25 most abundant taxonomical classifications across both experimental groups at all three sampling time points. Based on relative abundances, a change occurred between day 7 and day 21 as the dominant member, irrespective of experimental group, shifted from the genus *Lactobacillus* to *Faecalibacterium*. Minor differences in relative abundance of the genera *Lactobacillus*, *Bacterioidetes* and *Subdoligranulum* between groups were observed at day 7, 21 and 37, respectively. Beyond these, the control and probiotic group abundance tables for each sampling time point were very similar, and observable trends appeared over time, with little apparent influence from experimental group status.

**Fig. 5:**
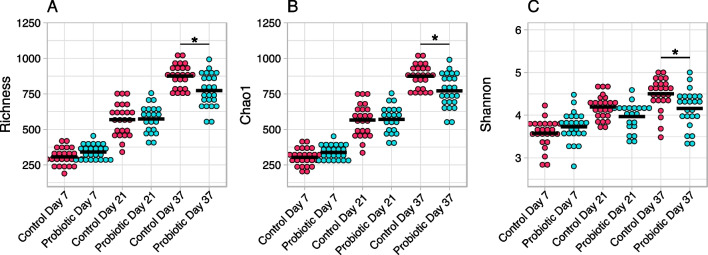
Sample specific microbiota diversity measures for each sample time point and both experimental groups in trial 1. **A** Richness, and **B** Chao1 and **C** Shannon diversity indices are shown. Group means are shown with a horizontal bar, overhead bars and asterisks denote statistically significant differences between groups, identified with a Kruskal–Wallis, followed by a pairwise Wilcoxon-test, using a Bonferroni-correction to correct for multiple comparisons. Relevant significant differences are indicated by asterisks as follows: * = *P* < 0.05

Next, the sample-specific alpha diversity, or complexity, was determined and compared between groups at each sampling time point by determining microbiota composition, both in terms of observed OTU’s and inferred OTU’s (Chao1), as well as Shannon diversity indices (Fig. 5).

Species richness (richness and Chao1), as well as complexity (Shannon) of the microbial communities was shown to generally increase over time. The only statistically significant differences between experimental groups, however, were found at day 37 (Richness: *P* = 0.04, Chao1: *P* = 0.04, Shannon: *P* = 0.02). The richness and Chao1 indices are virtually identical throughout, indicating a very limited influence of very-low abundance species on the richness of the cecal microbiotas, and the significant difference in Shannon index at day 37 suggests a significantly higher diversity in the control group.

**Fig. 6:**
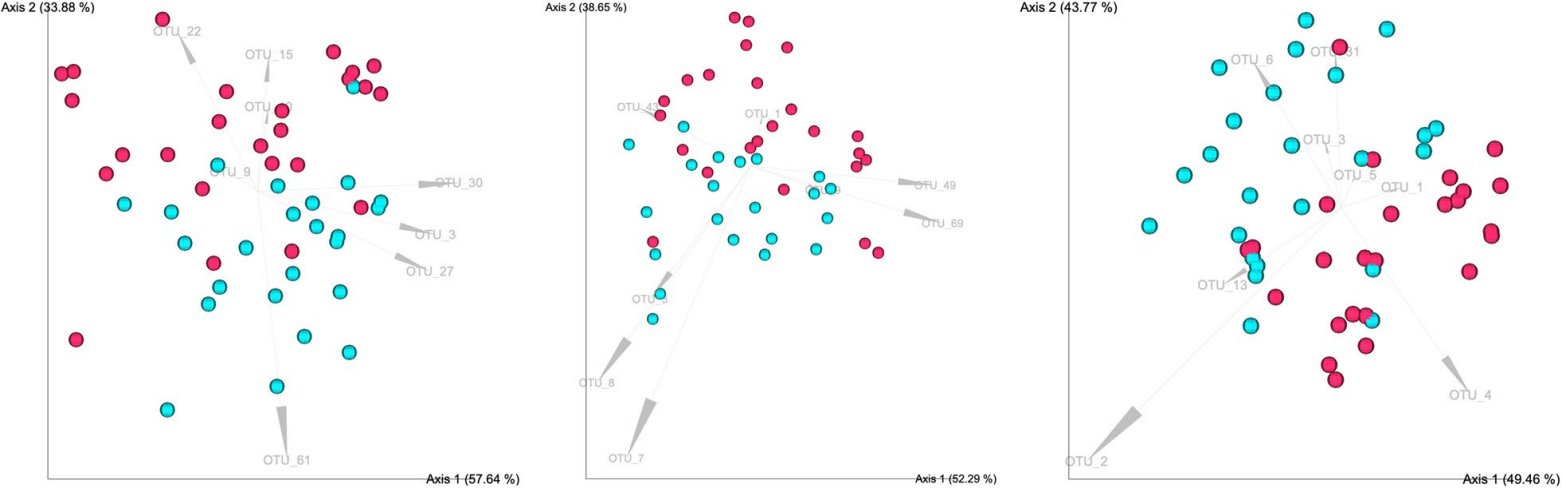
Beta diversity, visualized by robust principal component analysis (PCA) of cecal microbiota composition in trial 1. Individual plots created per each sampling time. **A** day 7, **B** day 21 and **D** day 37. Red and turquoise dots for control and probiotic treated birds, respectively

Finally, to describe compositional differences between samples, and to identify potential differences between treatment groups, a Robust Aitchison principal component analyses (RPCA) was performed using Aitchison distance of the 16S rRNA sequencing samples and is shown in Fig. 6. Arrows indicate prominent feature loadings on the distributions in each ordination plot. At each timepoint we find statistically significant microbiota composition clustering by treatment, given significant PERMANOVA testing and PERMDISP showing no significant difference in dispersion (See Fig. 6 and Table 3).

**Table 3:** PERMANOVA & PERMDISP testing, trial 1

Sampling point	PERMANOVA		PERMDISP
	Pseudo F-ratio	*P*-value	P-value
Day 7	12.79	0.001	0.17
Day 21	9.90	0.001	0.26
Day 37	12.26	0.001	0.44

## Discussion

In this study we investigated the effect of pre-hatch application of probiotic on production parameters and on the development of the intestinal microbiota of broilers from day 7 to 37. In terms of the probiotic treatment, the spray application method for mass administration proved successful as the probiotic strain could be consistently recovered from yolk sac, intestine and ceca for at least 7 days post hatch in both trials. During this period, a decreased recovery rate of the probiotic bacteria from the yolk sac and intestine of chickens was observed from day one to 7, and at day 21 the probiotic bacteria could no longer be recovered from ceca (trial 1). This indicates that the probiotic is transient in nature, and that it does not permanently colonize the intestine of the broilers after a single pre-hatch application.

Overall, the percentage of chickens colonized with probiotic in the second experiment was lower compared to the first experiment at day one (64 versus 96%, respectively) and day 7 (40 versus 46%, respectively). The actual sprayed concentration was slightly lower in trial 2 but it is unlikely that a lower cfu will result in fewer chickens to be colonized.

Following the application, hatchability was not affected by the spraying at day 18 of incubation. The hatchability of 84,6% in trial 1 fell within the expected range of 82–85% with 28-week-old parent birds. Also, in trial 2 the hatchability of 77,6% were within the expected range of 72–78% with 60 weeks old parent birds.

After hatching, first-week mortality differed in the two trials. In the first trial, 224 chickens died at day 3 in the control group and in total 386 died during the first week in this group. In the probiotic treated group only 129 chickens died during the first week. No known episode could explain the high mortality at day 3 in the control group. We found significant lower mortality in the probiotic treated group compared to the control group in the first trial, most likely due to the offset created between the two survival curves by the increased first-week mortality, but a similar difference was not observed during the second trial. Previous studies with the same strain of *E. faecium* showed that the strain protected against colonization with *S. enteritidis* and improved gut integrity [1]. It may be plausible that the probiotic treatment enhanced the gut integrity in broilers during first week, hereby mitigating the levels of pathogens, resulting in the observed lower mortality of the probiotic group during first week in trial 1. The effects of increased gut integrity may only be evident in the case the flock is challenged by pathogens. It may be the case that during trial 1 the flocks experienced a higher level of pathogens and thus we see a difference in mortality between the treated and untreated group. However, as these experiments were performed under commercial production conditions, we have no clear data to support the presence of a specific pathogen during the first week post hatch, and thus the influence of improved gut integrity remains speculative. Furthermore, the proportion of chickens which died due to infectious causes (mainly *E. coli*) in the treated and untreated group did not differ significantly and hereby does not support a hypothesis of improved resilience. Regardless of the cause and nature of the challenge, a significantly higher survival was observed in the probiotic group during trial 1.

The study is biased by the design where we use the same house for the probiotic treatment in both trial 1 and 2 and the same house for the control flock in both trials. This was done to limit the risk of cross-contamination of the probiotic bacteria from the treated flock in the first trial to the control flock in the second trial. However, we cannot disregard that a “house-factor” may impact the results, but this was preferred over the risk of cross-contamination of the control group in the second trial. Both production performance and microbiota development may be impacted by which house the flock were assigned to. When the houses for the study were selected, performance data from the 3 previous rotations were analyzed to see if there was a pattern in high or low performance regarding FWM, total mortality, FCR or bodyweight at slaughter. Since no consistent pattern could be observed the two houses were chosen for the trials.

In terms of production statistics, the FCR differs slightly between the two groups in trial 1, but EPEF was 9% higher in the probiotic treated group, reflecting the higher survival and higher body weight at day 34. The body weight differs by 5.4% (112 g) in favor of the probiotic treated group. In trial 2 the body weight and EPEF was 1.8% (38 g) and 2.3% higher in the probiotic compared to the control group. The performance parameters in general indicate a well driven farm with high production efficiency [9]. The difference in body weight at day 34 may be explained by lower burden of pathogens, resulting in fewer subclinical infections and hence better growth in the probiotic treated groups [10, 11]. Alternatively, it may be due to improved absorption of nutrients, which have been described in other trials explained by increased villi height resulting in a larger surface for nutrient absorption [10, 12, 13]. In a previous study using the same strain of *E. faecium* it was shown that the length of villi in jejunum increased after daily application of the probiotic strain [14]. Whether or not similar histo-anatomical effects are seen following pre-hatch spraying remains to be addressed in future evaluations of this application method.

The difference in production performance in trial 1 and 2 may be explained by biological variation or by the fact that fewer chickens were colonized with the probiotic bacteria in the second trial. The two trials were performed using the same farmhouses, which should help control for housing-related effects, but there are additional factors that could account for some of the observed variation. Thus, the results may have been affected by the different ages of the parent flock (28 versus 60 weeks). It has previously been described that parent age influences the body weight of broilers with higher parent age resulting in higher body weight in the broilers [15]. And the burden of pathogens in the environment may have differed between the two trials.

The microbiota analysis showed alpha diversities (Fig. 4) between 3.5–4.5. This range corresponds to what was found by Lundberg et al., (2021). Furthermore, the observed Shannon indices are in accordance with those observed for chicken cecal microbiotas when utilizing the V4 region of 16S rRNA, as reported in a recent meta-study [16]. Taken together the development in richness and diversity estimates throughout the timeframe that is the focus of the present study, suggest an increasingly rich and complex cecal microbiota. This is in line with published results from Ocejo *et al* [17], where species richness, Shannon indices, as well as relative abundance data demonstrate an increase in complexity of the cecal microbiota over time, in both broilers and free-range chicken. Clear, significant differences were finally observed when analyzing the between sample variation in microbiota composition, or beta diversity. While we cannot control for housing-related differences, these differences suggest a lasting effect of the probiotic application.

## Conclusions

Overall, the non-invasive prehatch application of *E. faecium* was successful in colonizing 96% and 64% of the chickens before/during hatch and could be re-isolated at day 7 although at a lower frequency. The production performance was positively impacted by the treatment in both trials as EPEF was increased compared to the control group in both trials.

Like a butterfly effect, pre-hatch application of *E. faecium* 669 positively affected production parameters as well as microbiota alpha and beta diversity measures beyond measurable retention in the chickens. Given the time of application, as well as the decreasing recovery of the probiotic strain during the first 21 days, it is noteworthy that significant effects on beta diversity are observed during and beyond this retention time on richness, and that significant differences in richness and alpha diversity measures are only observed beyond the last successful recovery of the probiotic. With a combination of relatively low-tech mass administration and promising performance, pre-hatch administration of *E. faecium* 669 by spray method should be considered a promising combination of methodology and probiotic for future studies and potential implementation.

## Material and methods

### Experimental setup

The effect of a probiotic *E. faecium* strain applied pre-hatch on the development of cecal microbiota and production performance of broilers under field conditions were evaluated. The trial was performed twice at a Danish hatchery and farm. Within each trial eggs for the control and the probiotic treated flock originated from the same parent flock. In trial 1 the parents were 28 weeks old while in the second trial the parent flock was 60 weeks old. The entire flocks (control and treated) serve as experimental unit in relation to production parameters. Regarding the cecal microbiota composition analysis which were performed on 25 randomly chosen broilers per flock, each bird serves as the experimental unit. The observational units were bodyweights, mortalities, FCR, EPEF, recovery ratios and concentrations of the probiotic strain from ceca and the cecal microbiota composition. The two experiments are considered genuine replicates [18]. The incubated eggs were sprayed with the probiotic solution when transferring from setters to hatchers at day 18 of incubation. One hatcher was used for probiotic sprayed eggs, and another was used for mock-sprayed eggs. Different trucks were used for transportation of the treated and untreated chickens to the farm. The newly hatched chickens were housed on the same farm in both experiments. To reduce the risk of cross-contamination between the two experiments the house used for control in the first experiment was also used for control in the second experiment and likewise for the probiotic treated. In trial 1, 22.000 and 21.000 chickens were included in the control and probiotic group, respectively while in trial 2, 24.100 and 24.200 were included in the control and probiotic group, respectively.

In trial 1, analysis of the microbiota development of ceca was conducted and 25 chickens from each group were collected randomly throughout the house and euthanized at day 7, 21 and 37. Ceca was removed by sterile scissors and tweezer, stored in sterile plastic, and transported on ice until storage at –20 °C before DNA extraction, library preparation and sequencing.

In addition, from both experiments 25 chickens per group were randomly collected and euthanized at day of hatch and day 7 (and 21 in trial 1) for recovery of the probiotic strain.

### Probiotic strain and application

Lyophilized *Enterococcus faecium* 669, 2 × 10^11^ colony forming units (cfu)/gram was used for pre-hatch application on fertilized Ross 308 eggs. In the following, the strain is designated *E. faecium.*

The *E. faecium* was applied at day 18 of incubation when eggs were transferred from the setter to the hatcher (Petersime, Zulte, Belgium). Two hatchers were used, one for control eggs and one for probiotic treated eggs. The hatchers had separate ventilation and contained 28.000 eggs each. All 28.000 eggs in each of the hatchers were sprayed with probiotic solution or sterile isotonic saline (mock spray).

The *E. faecium* was dissolved in sterile isotonic saline (37°C) for a final concentration of 1 × 10^10^ cfu/ml. In total 1.4 L probiotic solution was used for 28.000 eggs. The same amount of sterile isotonic saline was used for control eggs resulting in approximately 50 µl solution used per egg. A handheld pressure sprayer was used for the application.

After the spray process a sample of the probiotic solution was serially diluted in a 10-step tenfold dilution series (in triplicate). From the –7^th^ to –10^th^ dilution 100 µl were plated on blood agar plates (BA) prepared with 5% calf blood in blood agar base (Oxoid, Basingstoke, UK). The agar plates were incubated aerobically overnight at 37°C before colonies were counted for calculation of the actual cfu.

### Hatchery

The experiments were conducted at a commercial Danish hatchery. A standard hatching program was followed, and eggs were transferred from setters to hatchers at day 18 of incubation. Un-embryonated eggs were removed automatically after trans-illumination at the conveyer belt and probiotic spray was delivered after automatic transferring of the eggs from the setter trays to the hatching trays. Each hatching tray contained 73 eggs and eggs were laying on the side when sprayed.

### Recovery of the probiotic

The presence of the probiotic strain was investigated in the probiotic and the control group. From newly hatched chickens the yolk sac and intestine were used while only ceca were used from chickens day 7 and 21 for recovery of the *E. faecium* strain. The yolk sac, intestine and ceca were removed by sterile scissors and tweezers and equipment and gloves were changed for every chicken to avoid cross-contamination. Samples were homogenized in isotonic saline (1:1 ratio) using a Stomacher (Stomacher® 80, Seward, Lab systems) and serially diluted in a 4-step tenfold dilution series. For determination of the cfu/gram 10 µl of each dilution were plated in triplicate on blood agar plates with kanamycin (BA-kan) prepared with 5% calf blood in blood agar base (Oxoid, Basingstoke, UK) and supplemented with Kanamycin (1000 µg/ml) (Sigma-aldrich, Soeborg, Denmark). The agar plates were incubated aerobically at 37°C overnight before colony counts.

### Verification of the probiotic E. faecium by strain-specific PCR

For verification of the counted colonies on BA-kan plates one colony with typical morphology (pinpoint grey with beta-haemolysis) was re-streaked on a BA without kanamycin and incubated aerobically overnight at 37°C. Subsequently DNA was extracted by one of two methods: I) boiling lysate method: One loop (size of loop 1 µL) of bacterial colonies were suspended in 300 µl of miliQ water (vortex) and heated for 10 min at 100°C, then centrifuged for 5 min at 14,000 G before the supernatant was transferred to a new tube and used as DNA template. II) Extracted by use of Maxwell® RSC Instrument (Promega, Denmark) using Maxwell® RSC Cultured Cells DNA kit (AS 1620) (Promega, Denmark). Subsequently PCR was performed as previously described (Thøfner et al., 2021) with the primers shown in Table 4.

**Table 4:** Primer sequence and amplicon size for verification of *E. faecium* 669 probiotic strain

Primer name	Sequence (5′ → 3′)	Amplicon size (bp)
*E. faecium_F5*	*AGAACAGAGAAGTAGACCAGCCA*	920
*E. faecium_R5*	*TGAGGCTGCGATGTTGAAAGT*	

### Broiler farm

The control and probiotic treated flock in each experiment were housed on a large conventional broiler farm. The flocks were housed in separate but identical houses in regard of size, ventilation, bedding, temperature- and light-control, water system and feed. Before arrival, the houses were thoroughly cleaned and disinfected. The staff changed clothes, boots and gloves when entering the houses. Daily management were always completed in the control house before entering the probiotic treated house.

### Postmortem of chickens dead during first week of life.

All chickens that died during first week of life were collected at the farm and stored at − 20°C. A postmortem examination was performed on all chickens from the two flocks from day one to seven. Bacteriological sampling was done when macroscopic lesions indicated bacterial infection (increased vascularization, discoloration, exudations). The yolk sac or liver was sampled by a thin sterile cotton swab after sterilizing the surface with a hot iron. Samples were immediately plated on a BA and incubated aerobically overnight at 37°C. From plates showing dense growth of either presumptive *E. coli* (colony appearing as medium size, circular, convex, and greyish color) or *E. faecalis* (small, circular, convex and grey colonies) or both in mixture a single colony of presumptive *E. coli* or *E. faecalis* were subcultured on MacConkey agar (Oxoid, Basingstoke, UK) or bile aesculin agar (Oxoid, Basingstoke, UK), respectively. After overnight aerobic incubation at 37°C colonies on MacConkey agar with red/pink appearance were identified as *E. coli* and colonies on bile aesculin agar which colored the agar black were identified as *Enterococcus* spp..

### Production parameters

During production, first week mortality, the total mortality, feed conversion ratio, average body weight at day 7, 34 and at slaughter (day 37 and 38) was registered. At day 7 bodyweight was estimated based on FCR and visual appearance of chickens in the first trial while in the second trial 60 chickens from each house were weighed and the bodyweight was calculated as an average. The bodyweight at day 34 was likewise based on feed consumption while body weight at slaughter was based on data from the slaughterhouse.

Feed conversion ratio (FCR) was calculated based on the feed consumed divided by the average gained body weight. European production efficiency factor (EPEF) (Average grams gained per day x survival rate)/Feed conversion × 10) was used for comparing the production efficiency between the groups.

### Microbial community analysis of ceca

#### Library preparation and sequencing

From the euthanized chickens day 7, 21 and 37 (149 in total, also used for recovery of the probiotic strain) one ceca per chicken were stored at − 20°C before 16S ribosomal RNA sequencing was performed. DNA extraction, sequencing and quality control was performed by DNASense (https://dnasense.com/).

DNA extraction and sequencing library preparation was successful for 143 / 149 samples (96%) and yielded between 9203 and 187,855 DNA reads after QC and bioinformatic processing. Failed samples were those yielding significantly less quality filtered DNA reads than 10,000; here filtReads < 8,000. Low-read samples were disregarded in all subsequent analyses.

Sample DNA extraction was done using a slightly modified version of the standard protocol for FastDNA Spin kit for Soil (MP Biomedicals, USA) with the following exceptions. 500 μL of sample, 480 μL Sodium Phosphate Buffer and 120 μL MT Buffer were added to a Lysing Matrix E tube. Bead beating was done at 6 m/s for 4 × 40s [19]. Gel electrophoresis using Tapestation 2200 and Genomic DNA screentapes (Agilent, USA) was used to validate product size and purity of a subset of DNA extracts. DNA concentration was measured using Qubit dsDNA HS/BR Assay kit (Thermo Fisher Scientific, USA).

Archaea and Bacteria, 16S rRNA gene variable region V4 sequencing libraries were prepared by a custom protocol based on an Illumina protocol (Illumina, 2015). Up to 10 ng of extracted DNA was used as template for PCR amplification of the Archaea and Bacteria, 16S rRNA gene variable region V4 amplicons. Each PCR reaction (25 μL) contained (12.5 μL) PCRBIO Ultra mix and 400 nM of each forward and reverse tailed primer mix. PCR was done with the following program: Initial denaturation at 95° C for 2 min, 30 cycles of amplification (95° C for 15 s, 55°C for 15 s, 72° C for 50 s) and a final elongation at 72° C for 5 min. Duplicate PCR reactions were performed for each sample and the duplicates were pooled after PCR. The forward and reverse, tailed primers were designed according to (Illumina, 2015) and contain primers targeting the Archaea and Bacteria, 16S rRNA gene variable region V4: [515FB] GTGYCAGCMGCCGCGGTAA and [806RB] GGACTACNVGGGTWTCTAAT (Apprill et al., 2015). The primer tails enable attachment of Illumina Nextera adaptors necessary for sequencing in a subsequent PCR. The resulting amplicon libraries were purified using the standard protocol for CleanPCR SPRI beads (CleanNA, NL) with a bead to sample ratio of 4:5. DNA was eluted in 25 μL of nuclease free water (Qiagen, Germany). DNA concentration was measured using Qubit dsDNA HS Assay kit (Thermo Fisher Scientific, USA). Gel electrophoresis using Tapestation 2200 and D1000/High sensitivity D1000 screentapes (Agilent, USA) was used to validate product size and purity of a subset of sequencing libraries. Sequencing libraries were prepared from the purified amplicon libraries using a second PCR. Each PCR reaction (25 μL) contained PCRBIO HiFi buffer (1x), PCRBIO HiFi Polymerase (1 U/reaction) (PCRBiosystems, UK), adaptor mix (400 nM of each forward and reverse) and up to 10 ng of amplicon library template. PCR was done with the following program: Initial denaturation at 95° C for 2 min, 8 cycles of amplification (95° C for 20 s, 55° C for 30 s, 72° C for 60 s) and a final elongation at 72° C for 5 min. The resulting sequencing libraries were purified using the standard protocol for CleanPCR SPRI beads with a bead to sample ratio of 4:5. DNA was eluted in 25 μL of nuclease free water. DNA concentration was measured using Qubit dsDNA HS Assay kit. Gel electrophoresis using Tapestation 2200 and D1000/High sensitivity D1000 screentapes was used to validate product size and purity of a subset of sequencing libraries.

The purified sequencing libraries were pooled in equimolar concentrations and diluted to 2 nM. The samples were paired-end sequenced (2 × 300 bp) on a MiSeq (Illumina, USA) using a MiSeq Reagent kit v3 (Illumina, USA) following the standard guidelines for preparing and loading samples on the MiSeq. > 10% PhiX control library was spiked in to overcome low complexity issues often observed with amplicon samples.

### Quality control and analysis

Forward and reverse reads were trimmed for quality using Trimmomatic v. 0.32 (Bolger et al., 2014) with the settings SLIDINGWINDOW:5:3 and MINLEN: 225. The trimmed forward and reverse reads were merged using FLASH v. 1.2.7 (Magoč & Salzberg, 2011) with the settings -m 10 -M 250. The trimmed reads were dereplicated and formatted for use in the UPARSE workflow (Edgar, 2013). The dereplicated reads were clustered, using the usearch v. 7.0.1090 -cluster_otus command with default settings. Abundances of operational taxonomic units (OTUs) were estimated using the usearch v. 7.0.1090 -usearch_global command with -id 0.97 -maxaccepts 0 -maxrejects 0. Taxonomy was assigned using the RDP classifier [20] as implemented in the parallel_assign_taxonomy_rdp.py script in QIIME [21], using –confidence 0.8 and the SILVA database, release 132 [22]. All bioinformatic processing was done via RStudio IDE (1.2.1335) running R version 4.0.2 (2020–06-22) and using the R packages: ampvis (2.7.0) [19], tidyverse (1.3.0), seqinr (4.2.5), ShortRead (1.46.0), iNEXT (2.0.20) [23–25].

Rare OTUs were removed if abundance was lower than 0.001% (62 reads) of total assigned reads for Qiime2 analysis. Sample sizes after filtering ranged from 8242 to 18,7753 reads. Adequate sequencing depth for observing the vast majority of the microbiota after filtering was confirmed by rarefaction plots (See Additional file 1: Fig. S1) using Qiime2 [26].

Robust Aitchison distance between samples was calculated [27], visualized [28] and statistically tested using PERMANOVA & PERMDISP [29] for difference and dispersion between the two treatment groups using default values of Qiime2. Distances and statistical testing were done at each timepoint of sampling. Workflow and exact version of programs used during the Qiime2 analysis is available in Additional file 1: Fig. S2. The bioinformatic analyses were performed in collaboration with DNASense.

### Statistics

The results are presented as percentage (e.g., hatchability, recovery of probiotic, mortality etc.) and means with standard errors of mean (SEM) (cfu, body weight). For normalizing cfu counts, log10 transformation was applied. For recovery of probiotic, Prism 8 for Windows (version 8.4.0, GraphPad Software, Inc.) was applied for statistical analysis. Hellinger transformation and subsequent unrestricted principal component analysis of OTU compositions, as well as Kaplan–Meier plots of survival curves followed by comparison by log-rank was done in RStudio Version 1.4.1103 running R version 4.03 (2020–10-10), using the packages: survival (3.2–10) [30], survminer (0.4.9) (Kassambara et al., 2021), labdsv (2.0–1) [31] and readxl (1.3.1) [32]. An alpha level of 0.05 was set. A null-hypothesis stating that groups did not statistically significantly differ was abandoned at P-values < 0.05.

### Supplementary Information

**Additional file 1. Figure S1**. Rarefaction curves for all included cecal samples. Sample sizes after filtering range from 8242 to 187753 reads.

## Data Availability

Genomes will be uploaded and available from NCBI and programming scripts for microbiota analysis will be uploaded as supplementary data.
