# Supplementary material for: Effects of a novel, non-invasive pre-hatch application of probiotic for broilers on development of cecum microbiota and production performance
Source: Anim Microbiome. 2023 Sep 5;5:41. doi: 10.1186/s42523-023-00263-7 (PMC10478294; doi:10.1186/s42523-023-00263-7)
Supplement: Supplementary file 1 — Additional file 1. Figure S1. Rarefaction curves for all included cecal samples. Sample sizes after filtering range from 8242 to 187753 reads. [file 42523_2023_263_MOESM1_ESM.docx]

## Supplementary material.


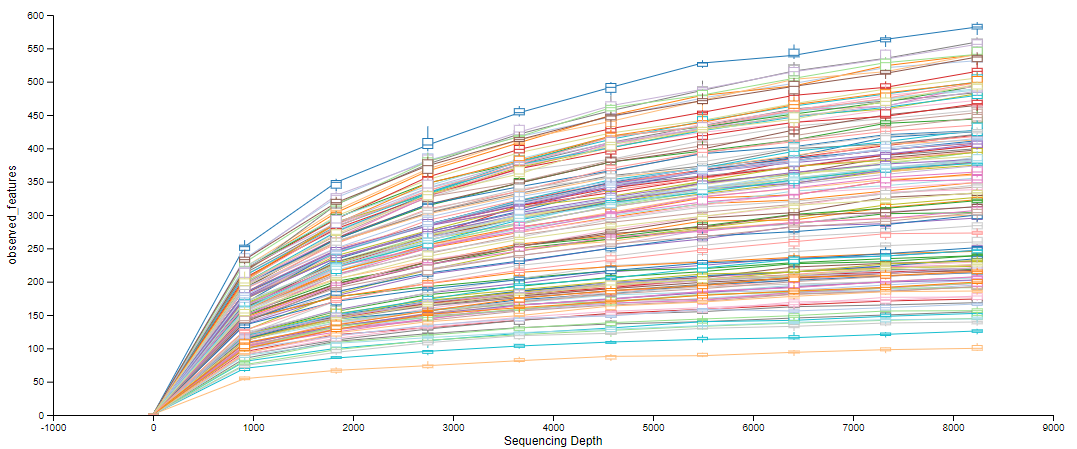


Figure S1. Rarefaction curves for all included cecal samples. Sample sizes after filtering range from 8242 to 187753 reads.
